# A virus associated with the zoonotic pathogen *Plasmodium knowlesi* causing human malaria is a member of a diverse and unclassified viral taxon

**DOI:** 10.1093/ve/veae091

**Published:** 2024-11-06

**Authors:** Mary E Petrone, Justine Charon, Matthew J Grigg, Timothy William, Giri S Rajahram, Jacob Westaway, Kim A Piera, Mang Shi, Nicholas M Anstey, Edward C Holmes

**Affiliations:** Sydney Infectious Diseases Institute, School of Medical Sciences, The University of Sydney, Sydney, NSW 2006, Australia; Laboratory of Data Discovery for Health Limited, 19 Science Park West Avenue, Hong Kong Science Park, Pak Shek Kok, New Territories, Hong Kong SAR, China; Fruit Biology and Pathology Unit, University of Bordeaux, INRAE, 71 Av. Edouard Bourlaux, Villenave-d’Ornon, Bordeaux 33140, France; Global and Tropical Health Division, Menzies School of Health Research, Charles Darwin University, John Mathews Building (Bldg 58), Royal Darwin Hospital Campus, Rocklands Drv., Casuarina, Darwin, NT 8010, Australia; Infectious Diseases Society Kota Kinabalu Sabah-Menzies School of Health Research Clinical Research Unit, Kota Kinabalu, Sabah 88200, Malaysia; Infectious Diseases Society Kota Kinabalu Sabah-Menzies School of Health Research Clinical Research Unit, Kota Kinabalu, Sabah 88200, Malaysia; Subang Jaya Medical Centre, No. 1, Jalan SS12/1A, Ss 12, Subang Jaya, Selangor 47500, Malaysia; Infectious Diseases Society Kota Kinabalu Sabah-Menzies School of Health Research Clinical Research Unit, Kota Kinabalu, Sabah 88200, Malaysia; Queen Elizabeth Hospital II, Ministry of Health Malaysia, Lorong Bersatu, Off, Jalan Damai, Luyang Commercial Centre, Kota Kinabalu, Sabah 88300, Malaysia; Global and Tropical Health Division, Menzies School of Health Research, Charles Darwin University, John Mathews Building (Bldg 58), Royal Darwin Hospital Campus, Rocklands Drv., Casuarina, Darwin, NT 8010, Australia; Global and Tropical Health Division, Menzies School of Health Research, Charles Darwin University, John Mathews Building (Bldg 58), Royal Darwin Hospital Campus, Rocklands Drv., Casuarina, Darwin, NT 8010, Australia; State Key Laboratory for Biocontrol, School of Medicine, Shenzhen Campus of Sun Yat-sen University, Sun Yat-sen University, Shenzhen 518107, China; National Key Laboratory of Intelligent Tracking and Forecasting for Infectious Diseases, Sun Yat-sen University, Shenzhen 518063, China; Shenzhen Key Laboratory for Systems Medicine in Inflammatory Diseases, Shenzhen Campus of Sun Yat-sen University, Sun Yat-sen University, Shenzhen 518063, China; Guangdong Provincial Center for Disease Control and Prevention, Guangzhou 510642, China; Global and Tropical Health Division, Menzies School of Health Research, Charles Darwin University, John Mathews Building (Bldg 58), Royal Darwin Hospital Campus, Rocklands Drv., Casuarina, Darwin, NT 8010, Australia; Infectious Diseases Society Kota Kinabalu Sabah-Menzies School of Health Research Clinical Research Unit, Kota Kinabalu, Sabah 88200, Malaysia; Laboratory of Data Discovery for Health Limited, 19 Science Park West Avenue, Hong Kong Science Park, Pak Shek Kok, New Territories, Hong Kong SAR, China; School of Medical Sciences, The University of Sydney, Sydney, NSW 2006, Australia

**Keywords:** RNA viruses, plasmodium, metagenomics

## Abstract

The Apicomplexa are a phylum of single-celled eukaryotes that can infect humans and include the mosquito-borne parasite *Plasmodium*, the cause of malaria. Viruses that infect non-*Plasmodium* spp. disease-causing protozoa affect the pathogen life cycle and disease outcomes. However, only one RNA virus (Matryoshka RNA virus 1) has been identified in *Plasmodium*, and none have been identified in zoonotic *Plasmodium* species. The rapid expansion of the known RNA virosphere via metagenomic sequencing suggests that this dearth is due to the divergent nature of RNA viruses that infect protozoa. We leveraged newly uncovered data sets to explore the virome of human-infecting *Plasmodium* species collected in Sabah, east (Borneo) Malaysia. From this, we identified a highly divergent RNA virus in two human-infecting *P. knowlesi* isolates that is related to the unclassified group ‘ormycoviruses’. By characterizing 15 additional ormycoviruses identified in the transcriptomes of arthropods, we show that this group of viruses exhibits a complex ecology as noninfecting passengers at the arthropod–mammal interface. With the addition of viral diversity discovered using the artificial intelligence–based analysis of metagenomic data, we also demonstrate that the ormycoviruses are part of a diverse and unclassified viral taxon. This is the first observation of an RNA virus in a zoonotic *Plasmodium* species. By linking small-scale experimental data to advances in large-scale virus discovery, we characterize the diversity and confirm the putative genomic architecture of an unclassified viral taxon. This approach can be used to further explore the virome of disease-causing Apicomplexa and better understand how protozoa-infecting viruses may affect parasite fitness, pathobiology, and treatment outcomes.

## Introduction

Parasitic protozoa are a highly diverse collection of single-celled eukaryotes that can cause disease in many vertebrates. Organisms belonging to the phylum Apicomplexa are associated with a range of human diseases including malaria (*Plasmodium*), inflammation of the brain (*Toxoplasma*) (Aguirre, 2019), diarrhoea (*Cryptosporidium*) (Ryan et al. 2014), and severe anaemia (*Babesia*) (Krause 2019). *Plasmodium* is the leading cause of death from the Apicomplexa in humans worldwide (Poespoprodjo et al. 2023). This mosquito-borne infection is estimated to have caused >240 million cases of malaria and to have killed >600 000 people in 2022 alone (WHO 2023).

Efforts to control and treat malaria are challenged by the complex ecology of this parasite (Lee et al. 2022) and mounting antimalarial drug resistance (Wellems and Plowe 2001, Rosenthal et al. 2024). Of the five human-only infecting species of *Plasmodium* (*P. falciparum, P. vivax, P. malariae, P. ovale wallikeri*, and *P. ovale curtisii*), *P. falciparum* and *P. vivax* cause the greatest morbidity and mortality, with *P. falciparum* accounting for >95% of malaria fatalities (Poespoprodjo et al. 2023). Partial resistance of *P. falciparum* to artemisinin is entrenched in the Greater Mekong subregion of Southeast Asia (Blasco et al. 2017, Ehrlich et al. 2020) and has now emerged independently in Africa (Conrad et al. 2023, WHO 2023, Rosenthal et al. 2024). Eight additional *Plasmodium* species can cause human malaria through zoonotic transmission via mosquito vectors (Fornace et al. 2023). Among these, *P. knowlesi* is the only species to cause severe disease and death in humans (Grigg et al. 2018, Rajahram et al. 2019, Anstey et al. 2021, Poespoprodjo et al. 2023). Although predominating in Malaysian Borneo (Cox-Singh et al. 2008, Cooper et al. 2020), *P. knowlesi* is now recognized as a significant cause of malaria across Southeast Asia (Tobin et al. 2024), in association with changing land use and deforestation (Grigg et al. 2017, Brock et al. 2019, Fornace et al. 2019, Tobin et al. 2024), and in areas with declining incidence of the cross-protective species, *P. vivax* (Anstey and Grigg 2019). Thus, innovative strategies are needed to combat and control *Plasmodium* as the efficacy of accessible treatments declines in the human-only species, and changes in land-use cause greater numbers of zoonotic malaria cases.

One potential approach for malaria control involves the use of viruses that infect disease-causing protozoa. In a similar manner to how bacteriophages have been leveraged to combat drug-resistant bacterial infections (Kortright et al. 2019, Hatfull et al. 2022, Strathdee et al. 2023), protozoa-infecting viruses have been proposed as a potential new avenue for therapeutics (Barrow et al. 2020, Zhao et al. 2023). These parasitic protozoan viruses (PPVs) (Zhao et al. 2023) have been identified in *Giardia* (Wang and Wang 1986), *Leishmania* (Grybchuk et al. 2018, Atayde et al. 2019), *Cryptosporidum* (Berber et al. 2021, Adjou et al. 2023), *Eimeria* (Revets et al. 1989, Ellis and Revets 1990, Roditi et al. 1994, Lee et al. 1996, Lee and Fernando 1998, 2000, Han et al. 2011, Wu et al. 2016, Xin et al. 2016), *Toxoplasma* (Gupta et al. 2024), *P. vivax* (Charon et al. 2019, Kim et al. 2019), and *Babesia* (Johnston et al. 1991, Hotzel et al. 1992). They are of particular interest because some impact the parasite life cycle and modulate disease outcomes in the parasite host. Notably, *Leishmania* species that harbour Leishmania RNA virus 1 have been associated with an increased risk of treatment failure in humans (Heeren et al. 2023) and more severe disease outcomes in mice (Atayde et al. 2019). Similarly, it has been proposed that infection of *Toxoplasma* with the recently characterized apocryptoviruses (*Narnaviridae*) may be associated with increased disease severity in humans (Gupta et al. 2024), although this has yet to be formally tested. Cryptosporidium parvum virus 1 modulates the interferon response in *Cryptosporidium*-infected mammals (Deng et al. 2023). To date, however, only one virus, Matryoshka RNA virus 1, has been identified in a *Plasmodium* species (*P. vivax*) (Charon et al. 2019, Kim et al. 2019), and it is not known whether this virus impacts *Plasmodium* fitness or disease pathogenesis in humans.

Extending the known diversity of PPVs requires innovative approaches to virus discovery because both protozoa and the viruses that infect them are likely ancient and often highly divergent. As a case in point, the ormycoviruses were first identified in parasitic single-celled eukaryotes and fungi using structure-based methods (Forgia et al. 2022) and have since been identified in kelp (Stramenopila) (Dekker et al. 2024), ticks (Martyn et al. 2024), palm (Niu et al. 2024), and additional fungal species (Pagnoni et al. 2023, Sahin et al. 2024). This group of bi-segmented RNA viruses shares no discernible phylogenetic relationship to known viral taxa, rendering it previously invisible to sequence-based discovery methods (Forgia et al. 2022). Little else is known about ormycoviruses including their complete host range or whether they encode positive- or negative-sense genomes. The application of artificial intelligence–based methods to metagenomic data (Hou et al. 2024), in addition to the large-scale sampling of aquatic environments (Zayed et al. 2022), has further uncovered previously inaccessible virus diversity, including entirely novel ‘supergroups’ of unclassified viral taxa (Hou et al. 2024). These tools and the data they have generated can be leveraged to explore the viromes of disease-causing protozoa including *Plasmodium*.

In this study, we combine these facets of virus discovery to characterize a divergent virus associated with human-infecting *P. knowlesi* isolates. We contextualize this virus within the vast viral diversity revealed through large-scale virus discovery studies. We also explore the complex ecology of viruses that infect parasites and can be transmitted as passengers to mammalian hosts (i.e., without directly infecting the mammal). Our findings extend the diversity of known *Plasmodium*-associated viruses and highlight the importance of integrating large- and small-scale virus discovery research to better understand viruses that infect these ancient, microscopic hosts.

## Results

### Identification of a divergent RNA virus associated with human-infecting *Plasmodium knowlesi*

To extend the known diversity of RNA viruses in disease-causing apicomplexans, we analysed the metatranscriptomes of 18 human blood samples with polymerase chain reaction (PCR)-confirmed *Plasmodium* infections and six uninfected human controls, collected in Sabah, east (Borneo) Malaysia between 2013 and 2014. These samples are the same as those previously described (Charon et al. 2019). Of the patients with malaria, seven were infected with *P. vivax*, six with *P. knowlesi*, and five with *P. falciparum* (Charon et al. 2019). Sequencing libraries were pooled according to *Plasmodium* species as were the negative controls, resulting in four libraries (SRR10448859–62, BioProject PRJNA589654). Matryoshka RNA virus 1 was previously found exclusively in all seven *P. vivax* isolates (SRR10448862) (Charon et al. 2019).

**Figure 1.**
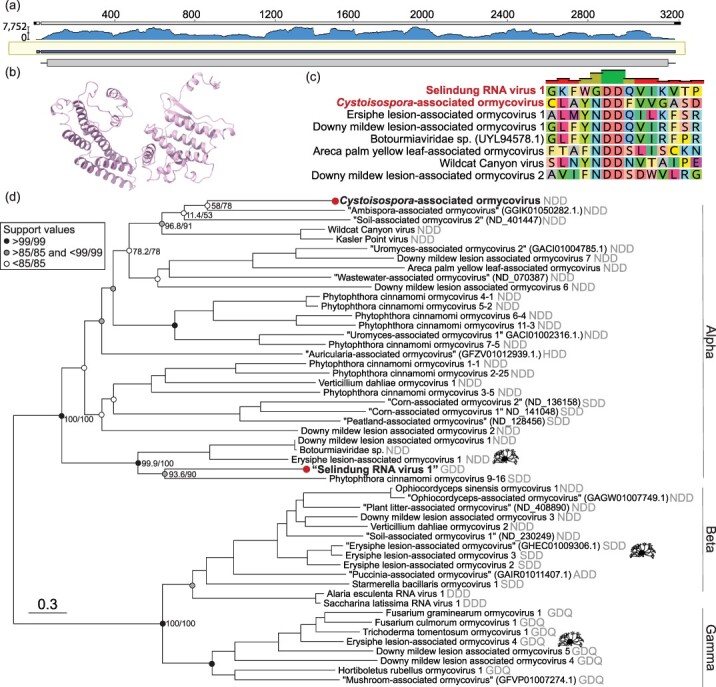
A divergent RNA virus associated with human-infecting *P. knowlesi* is a member of the unclassified group ‘ormycovirus’. (a) Sequencing coverage of the RdRp of a *P. knowlesi*-associated viral contig. Trimmed reads were mapped to the assembled contig using BBMap (Bushnell et al. 2017) and visualized with Geneious Prime v2024.0.7. (b) The predicted structure of the putative hypothetical protein of Selindung RNA virus 1. (c) MAFFT alignment of motif C in the palm domain of the *P. knowlesi*- and *Cystoisospora*-associated viruses and representative ormycoviruses. (d) Phylogenetic inference of the ormycoviruses aligned with MAFFT. The positions of *Erysiphe*-associated viruses are denoted with black icons (source: phylopic.org). Red tip dots indicate viruses identified in this study. Tips with names in quotes were previously identified but not named (Forgia et al. 2022). Their corresponding NCBI or “RNA Viruses in Metatranscriptomes database” (RVMT) accession is shown in parentheses. The catalytic triad encoded in each palm domain is denoted in grey. Support values are shown at select nodes as sh-aLRT/UFBoot. Tree branches are scaled to amino acid substitutions.

We searched each library for divergent viruses using the RNA-dependent RNA polymerase (RdRp)-scan bioinformatic pipeline (Charon et al. 2022). This revealed a putative, highly divergent RdRp that was 3177 nt in length with a complete open reading frame (ORF) and robust sequencing coverage (median coverage: 4004×) in the *P. knowlesi* library (SRR10448860) (Fig. 1a). No identical or related sequences were found in the other three libraries. The transcript was relatively abundant (1.4% of non-rRNA reads), and we confirmed the presence of this putative RdRp in two of the six isolates in the pool using reverse transcription PCR (RT-PCR) (Supplementary Table S1, Supplementary Fig. S1). Both patients with putative virus-infected *P. knowlesi* isolates were from Kota Marudu district residing in villages ∼30 km apart. There was a 3-month difference in the date of hospital presentation. Both had uncomplicated malaria with parasitaemia of 7177 and 41 882 parasites/µl, respectively, which were higher than the median parasitaemia found in the *P. knowlesi* infections that lacked the putative virus (4518/µl). Parasitaemia was correlated with the RdRp signals we observed with PCR (Supplementary Fig. S1).

Further inspection indicated that this putative virus was a bi-segmented ormycovirus likely infecting the *Plasmodium*. The divergent RdRp shared low but detectable sequence similarity with that of seven previously identified viruses, of which six were ormycoviruses (Supplementary Table S2). We identified a putative second segment of unknown function, 1721 nt in length sharing 22.8% identity (*e*-value = 3.14 × 10^−15^) with the hypothetical protein of Erysiphe lesion-associated ormycovirus 1 (USW07196). The structures of the putative and known hypothetical proteins were significantly similar (*P*-value = 1.62 × 10^−2^) when predicted with AlphaFold2 (Jumper et al. 2021, Mirdita et al. 2022) and compared by pairwise alignment with FATCAT (Li et al. 2020) (Fig. 1b). Similar transcripts were not identified in the ormycovirus-negative libraries from the same BioProject. Analysis of the library composition with CCMetagen (Marcelino et al. 2020) and the KMA database (Clausen et al. 2018) did not reveal plausible host candidates aside from the *Plasmodium*, which comprised 24% of non-rRNA reads. The remainder aligned to the *Hominidae*, reflecting that the *Plasmodium* were themselves infecting humans. We assumed that the host range of the ormycoviruses likely did not extend to vertebrates, consistent with their absence in humans without *Plasmodium* infection. Unlike its closest relatives, the *P. knowlesi*-associated RdRp encoded GDD in motif C of its palm domain rather than NDD (Fig. 1c).

To assess the prevalence of this and other ormycoviruses in *P. knowlesi*, we screened 1470 *P. knowlesi* RNA Sequence Read Archive (SRA) libraries (Supplementary Data S1) with a custom ormycovirus database. This returned no additional ormycovirus candidates. However, all 1470 libraries were generated from only seven BioProjects, and only the library we generated was derived from human-host *P. knowlesi* infections. The majority (*n* = 1356) were generated from macaque-host *P. knowlesi* infections, and all of these were generated by a single contributor from a small set of laboratory-maintained Rhesus macaques (PRJNA508940, PRJNA526495, and PRJNA524357). Sixty-one libraries were derived from cell culture, and the source of 52 (BioProject PRJEB24220) could not be determined. Thus, an accurate prevalence estimate of the *P. knowlesi*-associated ormycovirus could not be obtained from this data set.

We next investigated the prevalence of ormycoviruses more broadly in disease-causing apicomplexan by screening 2898 RNA SRA libraries (*Cryptosporidum, Coccidia, Toxoplasma, Babesia*, and *Theileria*) (Supplementary Data S2). This yielded identical ormyco-like RdRp segments in the transcriptomes of 22 Coccidia (*Cystoisospora suis*) libraries, 21 of which belonged to the same BioProject (PRJEB52768) (Cruz-Bustos et al. 2022). The remaining library (SRR4213142) was published by the same authors, suggesting that all 22 libraries were generated from the same source (Palmieri et al. 2017). This bias precluded us from inferring the prevalence of ormycoviruses in *Cystoisospora*. The transcripts of the *Cystoisospora*-associated virus encoded complete ORFs with an NDD motif C and were ∼3.1 kb in length (range: 3009–3203) (Supplementary Table S3). This virus was highly divergent, sharing only 32.5% identity (*e*-value = 6 × 10^−37^) with its closest blast hit (Wildcat Canyon virus, WZL61396.1). It was also at low abundance across the 22 libraries (range: 0.01%–0.08% of non-rRNA reads). We could not conclude that *C. suis* was the host because fungi represented 4.6% of the non-rRNA reads in a representative library (ERR9846867). Regardless, the prevalence of ormycoviruses was 100% among *Cystoisospora suis* libraries but otherwise very low in this data set (0.76%).

Phylogenetic analysis placed both apicomplexan-associated viruses in the ‘Alpha’ clade of the ormycoviruses (Fig. 1d). The topology of the inferred phylogenies was stable across six combinations of alignment and trimming methods and recapitulated the three main ormycovirus clades ‘Alpha’, ‘Beta’, and ‘Gamma’ (Forgia et al. 2022) with strong support (Supplementary Fig. S2). Viruses did not cluster by host. For example, viruses associated with the fungal species *Erysiphe* fell across all three clades and encoded three different catalytic triads (Fig. 1d, ‘icons’), and the apicomplexan-associated viruses were not closely related within the Alpha clade.

We concluded that the *P. knowlesi*-associated virus represents the first evidence of an RNA virus associated with *P. knowlesi* and constitutes only the second instance of an RNA virus associated with any *Plasmodium* species. We have provisionally named it ‘Selindung RNA virus 1’ because it appeared to be concealed (‘terselindung’, Bahasa Malaysia) within the *Plasmodium* parasite, and we will use this name herein.

### Ormycoviruses are associated with arthropod metatranscriptomes

In addition to expanding the diversity of *Plasmodium*-associated RNA viruses, Selindung RNA virus 1 was of particular interest because it had evidently been transmitted along with its *Plasmodium* host to a human via a mosquito vector. Taking this together with the detectable phylogenetic relationship of this virus and two viruses recovered from tick metagenomes (Wildcat Canyon virus and Kasler Point virus), we posited that ormycoviruses might exhibit a nested ecology at the arthropod–mammal interface. We therefore sought to further extend the known host range of ormycoviruses to the transcriptomes of the arthropods that indirectly transmit them.

We screened the 4864 arthropod libraries available on NCBI Transcriptome Shotgun Assemblies (TSAs) as of August 2024, initially using Kasler Point virus (WZL61394) as input and then following an iterative process (see Methods). In this way, we identified 15 putative viruses associated with three of the four extant subphyla of the Arthropoda: Chelicerata (*n* = 1), Crustacea (*n* = 1), and Hexapoda (*n* = 13) (Supplementary Table S4). All shared detectable but minimal sequence similarity with published ormycoviruses (range: 27.1%–41.0%, Supplementary Table S4). Two encoded GDD at motif C like Selindung RNA virus 1, while the remainder had NDD at this position.

**Figure 2.**
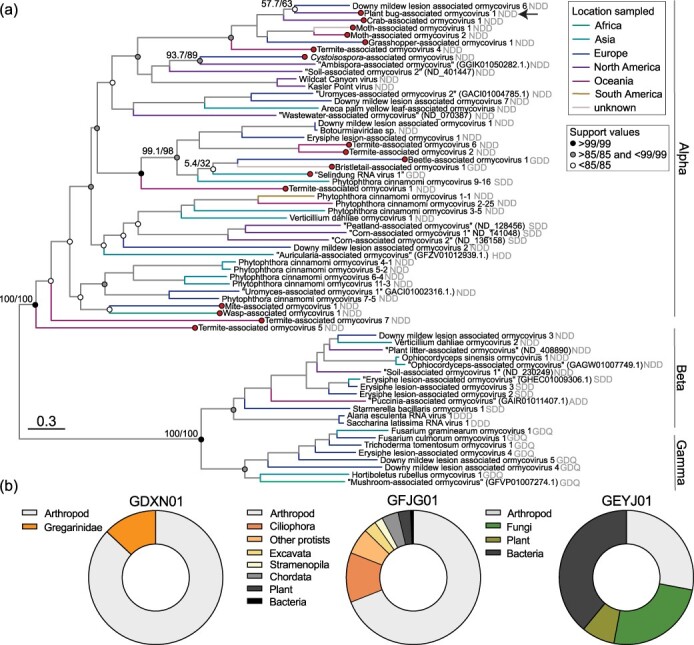
Ormycoviruses are associated with arthropod transcriptomes. (a) Phylogenetic inference of the extended diversity of ormycoviruses. Viruses identified in this study are indicated by red circles. The arrow indicates the position of the virus that appears to use the ciliate genetic code. Clades are annotated according to designations established by Forgia et al. (2022). The catalytic triad encoded in each palm domain is denoted in grey. The tip labelling scheme for unnamed viruses (denoted by quotation marks) is the same as in Fig. 1. Support values are shown at select nodes as sh-aLRT/UFBoot. Tree branches are coloured by the location where each tip was sampled, and they are scaled by amino acid substitutions. (b) Library composition of select arthropod assemblies. The graph labels correspond to the TSA project ID.

Phylogenetic analysis again supported the conclusion that these viruses are part of the ormycovirus group (Fig. 2a). All viruses identified in this study fell in the Alpha clade. Selindung RNA virus 1 formed a group with the two other viruses that encode GDD at motif C in the palm domain of the RdRp (Beetle-associated ormycovirus 1 and Bristletail-associated ormycovirus 1). This placement was consistent across all six iterations of phylogenetic inference (Supplementary Fig. S3). However, aside from this instance and the Gamma clade (GDQ motif), minimal clustering of motifs was observed. In addition, although the host organisms had been collected from all six inhabited continents, there was no clustering of viruses by geographic region of sampling, perhaps a reflection of the unrealized diversity and ancient evolutionary history of this taxon (Fig. 2a).

We concluded that these viruses were likely infecting fungi and single-celled organisms rather than the arthropods themselves for three reasons. First, the assessment of each library composition revealed instances of parasitic hosts. Contigs mapping to alveolates accounted for more than one-tenth of one Hexapoda (GDXN01) and the only crustacean (GFJG01) library (13% *Gregarinidae* and 12% Ciliophora, respectively) (Fig. 2b). Similarly, the mite assembly (GEYJ01) included 25% of contigs mapping to fungi, which are known hosts of ormycoviruses (Fig. 2b). Second, the virus identified in GBHO01 (*Lygus hesperus*) likely utilized the ciliate genetic code (i.e. only a truncated ORF could be recovered with the standard genetic code) yet fell within the diversity of the taxon (Fig. 2a, Supplementary Fig. S3, ‘arrow’). Thus, we assumed that any organism that utilizes the standard genetic code was unlikely to be the host of this putative virus. Identical amino acid translations of the crustacean-associated virus were produced when either the standard or the ciliate genetic code was used, rendering the ciliates detected in these libraries as plausible hosts. Third, the phylogenetic placement of arthropod-associated ormycoviruses was interspersed among known fungal and protozoan-infecting ormycoviruses, suggesting that animals were unlikely to be the host of the newly identified viruses (Fig. 2a). We therefore concluded that nonanimal eukaryotes are the most likely hosts of the ormycoviruses.

As with the *P. knowlesi* library, we searched these assemblies for hypothetical proteins. From this, we identified a putative second segment in the *Machilis pallida* (Hexapoda) assembly HBDP01 containing Bristletail-associated ormycovirus 1 that was 1619 bp in length and encoded a partial ORF (HBDP01002991.1). We could not recover candidates corresponding to the remaining libraries or assemblies.

### The ormycoviruses are members of a diverse and unclassified viral taxon

The wide host range of the ormycoviruses, spanning Alveolata, Stramenopila, and Opisthokonta (Fungi), suggested that this unclassified group harboured unrealized viral diversity. We therefore aimed to contextualize the diversity of the ormycoviruses within unclassified taxa identified in virus discovery studies. To do this, we assembled a custom database of viruses that were identified with an artificial intelligence-based analysis of metagenomic data (Hou et al. 2024) and screened the ormycoviruses against it using DIAMOND Blastx (Buchfink et al. 2021). This approach placed ormycoviruses within an unclassified taxon referred to in the original study as the proposed ‘SuperGroup 024’ (Hou et al. 2024), a name which we will use herein.

**Figure 3.**
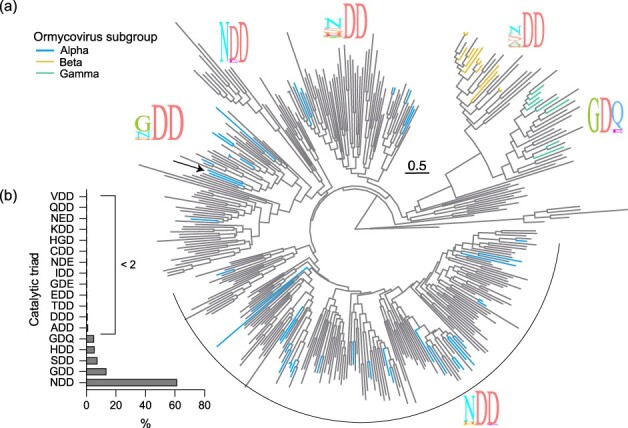
Ormycoviruses are members of a diverse and unclassified viral taxon with a flexible motif C in its RdRp palm domain. (a) Phylogenetic inference of viruses in SuperGroup 024 (Hou et al. 2024). Branches are coloured by their placement in the ormycovirus-only phylogenetic tree (Figs 1 and 2). Grey tree branches indicate that those tips were not previously recognized as ormycoviruses. The icons show the proportion of individual amino acids at each position of the catalytic triad in motif C of the RdRp palm domain for the corresponding clades. The arrow indicates the topological position of Selindung RNA virus 1. Tree branches are scaled according to amino acid substitutions. (b) Distribution of catalytic triads encoded by members of SuperGroup 024. The *x*-axis shows the percentage that each triad comprises among all known SuperGroup 024 species.

Phylogenetic analysis illustrated that the current set of ormycoviruses represent only a fraction of the total diversity of this group as they fell throughout the phylogeny. Interestingly, the addition of the SuperGroup 024 viruses expanded the diversity of the Alpha group, scattering the original members across three sections of the tree (Fig. 3a, ‘blue branches’). The Beta and Gamma clades were unchanged and characterized by a long branch at their shared base (Fig. 3a, ‘green and yellow branches’).

Members of SuperGroup 024 encoded a more diverse set of catalytic triads at the motif C palm domain compared to the original ormycovirus data set (Forgia et al. 2022) (Fig. 3b). However, their addition did not lead to observable clustering of discrete motif sequences, as flexibility was observed throughout the phylogeny. Selindung RNA virus 1 again fell in a section predominated by GDD at that position (Fig. 3a, ‘arrow’).

We searched the libraries containing SuperGroup 024 RdRp segments for ormycovirus hypothetical proteins. Of the 259 SRA libraries in which SuperGroup 024 RdRps were detected and assembled, we recovered hypothetical protein candidates at least 1000 bp in length in 190 (73.4%). It was not possible to assign hypothetical proteins to corresponding RdRps as many libraries contained multiple RdRp segments. Despite this, our finding supports the conclusion that bisegmentation is a characteristic of viruses in this taxon and that ormycoviruses and SuperGroup 024 are one and the same.

## Discussion

This study expands the diversity of *Plasmodium*-associated RNA viruses and presents the first evidence of an RNA virus associated with zoonotic transmission of *P. knowlesi*. Previously, only Matryoshka RNA virus 1 (*Narnaviridae*) had been identified in a *Plasmodium* species (the human-only *Plasmodium* species, *P. vivax*) (Charon et al. 2019, Kim et al. 2019). Although it is not possible to conclusively establish that Selindung RNA virus 1 was infecting *P. knowlesi* from metatranscriptomic data alone, lines of indirect evidence suggest that it was. Most notably, no other probable hosts, including fungi, were identified in the library, and the RdRp contig was relatively abundant (1.4% of non-rRNA reads). Contamination was an unlikely source because neither the putative RdRp nor the second segment was detected in the other three libraries extracted and sequenced at the same time. In addition, we were able to confirm the presence of the RdRp segment in two of the six *P. knowlesi* isolates using PCR. We therefore concluded that Selindung RNA virus 1 most likely represents an RNA virus in a second *Plasmodium* species.

Robust sampling of natural *P. knowlesi* infections is needed to evaluate the prevalence and pathobiology of Selindung RNA virus 1. We observed one instance of Selindung RNA virus 1 among 1470 SRA libraries, which suggests that associations occur infrequently and contrasts with the identification of Matryoshka RNA virus 1 in 13 of 30 *P. vivax* SRA libraries (Charon et al. 2019). However, ours was the only library to have been generated from isolates collected from naturally infected humans, while most of the publicly available data were derived from laboratory experiments. The detection of the RdRp segment in two of six isolates in our library could indicate that associations are more frequent in natural infections in Sabah, but our study was not sufficiently powered to address this. Similarly, whether the observation that the presence of the virus was correlated with higher parasitaemia is meaningful requires further epidemiological investigation.

Arthropods are a powerful tool for measuring the prevalence of viruses in nature, particularly when sampling from humans or other vertebrates is not feasible. The identification of ormycoviruses in arthropod metatranscriptomes and in a human blood sample suggests that these viruses represent a unique type of arbovirus that can be transmitted as a passenger between arthropods and mammals. Mosquito-based surveillance methods have been proposed for tracking the incidence and spread of human pathogens (Grubaugh et al. 2015, Fauver et al. 2018). Unlike cell culture or primary samples, which rely on symptomatic individuals with access to diagnostic testing, arthropod-based surveillance would be relatively unbiased, enabling more accurate estimates of protozoan virus prevalence and diversity within communities. When combined with cell culture data, this approach could also be used to parse arthropod- and protozoan-infecting viruses. Because they can be indirectly transmitted by arthropods, it may be that other protozoan viruses have already been identified, but their relationship to their protozoan host was obscured because they were part of an arthropod metatranscriptome.

An incidental and surprising finding was the identification of an ormycovirus that appears to use a nonstandard genetic code (Plant bug-associated ormycovirus 1). Despite this difference, the virus fell within the diversity of the ormycoviruses and SuperGroup 024. As RNA viruses are reliant on host machinery for translation, it was previously proposed that the evolution of alternative genetic codes was an antiviral defence (Shackelton and Holmes 2008). Under this assumption, the use of host-specific genetic codes by RNA viruses would imply a long-term virus–host coevolutionary relationship, and we would not expect to find viral taxa in which members use different genetic codes. Genetic code switching has been observed infrequently in the *Picornavirales* and *Lenarviricota* (Chen et al. 2022). Whether these few instances are an aberration in an otherwise broadly held rule of virology requires further investigation. However, we posit that there may be many more instances of code switching within known viral taxa that have been overlooked as a consequence of inadequate bioinformatic workflows. For example, if we had used an automated pipeline that filtered out contigs that did not produce an ORF with the standard genetic code, Plant bug-associated ormycovirus 1 would have been removed from our data set. We therefore advocate for the inclusion of multiple genetic codes when searching for divergent RNA viruses.

That Selindung RNA virus 1 does not belong to a classified viral taxon is notable because it demonstrates that parasitic protozoa likely harbour currently unrealized diversity, and additional discoveries may be imminent as new bioinformatic tools are developed to explore the RNA virosphere. However, the discovery of the ormycoviruses highlights the importance of linking large-scale metatranscriptomic data to smaller-scale experimental work when searching for protozoan viruses. Large-scale virus discovery studies often prioritize environmental samples such as water (Zayed et al. 2022), sediment (Hou et al. 2024), and soil (Chen et al. 2022) because these biodiverse sources are rich with RNA viruses. Yet, this approach cannot distinguish between bacteria-, archaea-, and eukaryotic-infecting RNA viruses. Without the discovery of the ormycoviruses and the experimental validation by Forgia et al. (2022), SuperGroup 024 would have been overlooked as a potential source of protozoan virus candidates. Similarly, large-scale studies are not equipped to distinguish segmented from nonsegmented viruses because they necessarily focus on detecting RdRps, rendering them ‘blind’ to segmentation. The molecular characterization of the ormycoviruses again demonstrates this limitation because their hypothetical protein does not share detectable sequence or structural similarity with known viral proteins.

This, as with other metagenomic studies, primarily serves to generate hypotheses and raise questions about RNA virus evolution and biology that require additional experimental data to answer. It is not known whether the ormycoviruses are positive- or negative-sense viruses. Forgia *et al*. observed a higher proportion of negative-sense RNA in their samples but could not draw a definitive conclusion in the absence of a true virion (Forgia et al. 2022). The presence of both SDD and GDD catalytic triads in motif C of the palm domain counters the hypothesis that SDD is specific to segmented negative-sense RNA viruses (Venkataraman et al. 2018), although it is possible that ormycoviruses do indeed fall into this category. The flexibility of the catalytic triad also raises the question of whether individual triads have a detectable impact on the biology of the virus and why flexibility is permitted in an otherwise highly conserved region of the virus genome. From a global health perspective, the most important questions to address include how viral infection of *Plasmodium* affects onward *Plasmodium* transmission and the pathobiology of *Plasmodium* in humans. Additionally, which part of the parasite the virus infects and whether this could be used as a potential drug target remain unanswered. It has already been shown that viruses can serve as a weapon against drug-resistant bacterial infections (Kortright et al. 2019, Hatfull et al. 2022, Strathdee et al. 2023). Whether a similar approach could be deployed to combat malaria and other disease-causing apicomplexan should be a research priority.

## Methods

### Human malaria isolates

*Plasmodium* RNA was isolated from cryopreserved red cells collected from 18 patients with acute malaria, enrolled in Kudat Division, Sabah, Malaysia in 2013 and 2014 (Grigg et al. 2018). PCR was used to confirm *Plasmodium* species as *P. knowlesi* (*n* = 6), *P. vivax* (*n* = 7), and *P. falciparum* (*n* = 5). The methods for species typing and RNA extraction were reported previously (Charon et al. 2019).

### SRA library data sets

#### BioProject PRJNA589654 libraries

*Plasmodium* SRA libraries in BioProject PRJNA589654 (*n* = 4) (i.e. the BioProject that contained Matryoshka RNA virus 1) were downloaded from NCBI. Nextera adapters were trimmed using Cutadapt v.1.8.3 (Martin 2011) with the parameters removing five bases from the beginning and end of each read, a quality cut-off of 24, and a minimum length threshold of 25. The quality of trimming was assessed using FastQC v0.11.8 (Andrews 2023). rRNA reads were removed using SortMeRNA v4.3.3 (Kopylova et al. 2012), and non-rRNA reads were assembled using MEGAHIT v1.2.9 (Li et al. 2015).

#### Disease-causing apicomplexan libraries

We downloaded all *P. knowlesi* RNA SRA libraries of at least 0.5 Gb in size available on NCBI as of August 2024 (*n* = 1470). We also downloaded all RNA SRA libraries for *Cryptosporidium*, *Coccidia*, *Toxoplasmosis*, *Babesia*, and *Theileria* available on NCBI as of March 2024 that are at least 0.5 Gb in size and generated on the Illumina platform (*n* = 3162).

#### SuperGroup 024 libraries

To analyse the libraries containing RdRp segments of so-called SuperGroup 024 (Hou et al. 2024), we first downloaded all of the contigs designated in this group by Hou et al. (2024) (http://47.93.21.181/). We then extracted the corresponding SRA libraries from each sequence header and removed duplicates (*n* = 273). All but one were downloaded from NCBI. The library SRR1027962 failed repeated attempts to download, likely due to its size (99.8 Gb).

### Arthropod TSA screen

We began by screening all arthropod TSA (*n* = 4864) available in August 2024, using Kasler Point virus (a tick-associated ormycovirus) as input. This screen was performed with tBLASTn implemented in the NCBI Blast web interface (https://blast.ncbi.nlm.nih.gov/Blast.cgi). All hits were reviewed and filtered according to three criteria: (I) the contig was at least 800 bp in length, (II) the contig encoded an uninterrupted ORF, (III) the contig did not return any hits to cellular genes when screened against the NCBI nonredundant (nr) database. We then aligned our filtered data set using MAFFT (Katoh and Standley 2013) with default parameters and selected the most divergent virus according to the distance matrix. This virus was then used as input for an additional screen of the arthropod TSA. This process was repeated until no new contigs were identified.

### Library processing

#### Contig assembly

For all data sets obtained from the SRA, Nextera adapters were trimmed using Cutadapt v.1.8.3 (Martin 2011) with the parameters described earlier. The efficacy of trimming was assessed using FastQC v0.11.8 (Andrews 2023). In total, 1470 *P. knowlesi* libraries, 2898 additional apicomplexan libraries, and 259 libraries included by Hou et al. (2024) were successfully assembled using MEGAHIT v1.2.974.

#### Abundance estimates

The expected count of putative viral transcripts was inferred using RSEM v1.3.0 (Li and Dewey 2011). For the *P. knowlesi* library containing the ormycovirus (SRR10448860), reverse-strandedness was specified to match the sequencing protocol. Default parameters were used for the remaining libraries. To infer the proportion of reads of each putative viral transcript, we calculated the total expected count for the isoforms in each library and used this value as the denominator to measure the percentage that putative viral reads comprised in the library. This analysis was performed using R v4.4.0.

### Identification of divergent viruses

#### Polymerase segment identification

We identified Selindung RNA virus 1 using the RdRp-scan workflow (Charon et al. 2022). Briefly, we screened the protein sequence and Hidden Markov Models (HMM) profile of assembled contigs from each library against a viral RdRp database. To search for additional divergent viruses, we screened all SRA libraries against the RdRp-scan database (Charon et al. 2022) and a custom database containing known ormycoviruses using DIAMOND Blastx v2.0.9 (Buchfink et al. 2021) and the setting ‘ultra-sensitive’. This database included the 39 published ormycoviruses and the Selindung RNA virus 1 RdRp segment. Only hits with *e*-values <1e-07 were retained for further analysis. Contigs with hits to this database were then screened against the NCBI nr protein database to remove false positives, again using DIAMOND Blastx v2.0.9 (Buchfink et al. 2021) and an *e*-value threshold of 1e-07. The parameter ‘very-sensitive’ was specified. Contigs that shared detectable sequence similarity to cellular genes were excluded from further analysis. Nucleotide sequences were translated using Expasy (https://web.expasy.org/translate/). The standard genetic code was used by default. Contigs that did not return an ORF in any frame with this code were checked manually using all codes available in Expasy.

#### Second segment identification

We first used blastn to screen libraries for contigs sharing conserved 5ʹ and 3ʹ termini of the corresponding ormycovirus RdRp. When this did not reveal any candidates, we compiled a database of all known ormycovirus second segments and used this to screen all SRA libraries using DIAMOND Blastx v2.0.9 (Buchfink et al. 2021). Contigs that had statistically significant hits to this database were checked against the NCBI nr protein database to remove false positives (i.e. nonendogenous viral element cellular genes). Nucleotide sequences were either translated individually with Expasy (https://web.expasy.org/translate/) or with InterProScan v5.65-97.0. For sequences processed with the latter, the longest translated ORFs were used for downstream analysis. To tally the number of SuperGroup 024 libraries with detectable hypothetical proteins, we cross-checked the presence of RdRp segments and hypothetical protein segments in each library using R v4.4.0.

For the primary *P. knowlesi* library, we searched for similar sequences to those at the 5ʹ and 3ʹ termini of the RdRp segment in other contigs in the library. To do this, we extracted these regions from the RdRp segment and used each as input for tblastn against the assembled library (SRR10448860). To ensure that the putative Selindung RNA virus 1 hypothetical protein was not present in other libraries in the same BioProject, we used this sequence as input for tblastn against the three remaining libraries.

Both tblastn screens were implemented in Geneious Prime v2024.0.7 and default parameters were used.

#### PCR validation

We first generated cDNA from the isolates using the SuperScript IV reverse transcriptase (Invitrogen). These products were then used as templates for amplification with PCR. Reactions were carried out in a total volume of 50 µl, of which 25 µl was the SuperFi II (Invitrogen) master mix and 1 µl was the cDNA template. A volume of 2.5 µl of forward and reverse primers was used (Supplementary Table S1). Reactions were performed on a thermocycler with the following conditions: 98°C for 1 min followed by 35 cycles of 98°C for 10s, 60°C for 10s, 72°C for 1 min, and 72°C for 5 min. The PCR products were analysed on an agarose gel. We used *Plasmodium* LDHP primers as the positive control.

### Library composition analysis

#### CCMetagen

The composition of individual sequencing libraries was assessed using ccmetagen v1.2.4 (Marcelino et al. 2020) and kma v1.3.9a (Clausen et al. 2018) with assembled contigs as input. The results presented in Fig. 2b were visualized with Prism v.10.3.0.

### Protein structure inference

The structure of the putative hypothetical proteins of Selindung RNA virus 1 and Erysiphe lesion-associated ormycovirus 1 was predicted using AlphaFold2 (Jumper et al. 2021, Mirdita et al. 2022) implemented in the Google Colab cloud computing platform. The confidence (as measured by pLDDT) of the prediction was compared across five models, and the highest performing models (Selindung RNA virus 1: #2, Erysiphe lesion-associated ormycovirus 1: #4) were selected for downstream analysis (Supplementary Fig. S4). To assess structural similarity, we performed a pairwise alignment of the resulting PDB files of each predicted structure using FATCAT (Li et al. 2020). All PDB files were visualized in ChimeraX v1.7.1 (Meng et al. 2023).

### Functional domain inference

Several approaches were used to infer functional domains in the hypothetical protein, although none were successful. We first performed a preliminary check with InterProScan (Jones et al. 2014), screening against the CDD, NCBIfam, and TMHMM databases. This approach was implemented in Geneious Prime v2024.0.7. We then employed Phyre2 (Kelley et al. 2015) and HHPred (Zimmermann et al. 2018) using PDB. Finally, we used the predicted structure of the hypothetical protein of Selindung RNA virus 1 as input for FoldSeek (van Kempen et al. 2023), implemented on the Foldseek Server.

### Phylogenetic analysis

To assess the phylogenetic relationships of the ormycoviruses identified in this study with those documented previously, we compiled a data set of all known ormycoviruses. This comprised 36 ormycoviruses (Forgia et al. 2022, Pagnoni et al. 2023, Dekker et al. 2024, Martyn et al. 2024, Niu et al. 2024, Sahin et al. 2024) and unclassified or misclassified ormycoviruses that shared detectable sequence similarity with known ormycoviruses: Wildcat Canyon virus (WZL61396), Kasler Point virus (WZL61394), and a fungus-associated ‘Botourmiaviridae’ (UYL94578). For the SuperGroup 024 analysis, we utilized the data set featured in the phylogenetic analysis presented by Hou et al. (2024).

We first added the *P. knowlesi*-associated and *Cystoisospora*-associated viruses identified in this study to the ormycovirus data set and aligned with MAFFT v7.490 (Sievers et al. 2011) and MUSCLE v5.1 (Edgar 2022). Ambiguities in each alignment were considered in three ways using trimAl v1.4.1 (Capella-Gutiérrez et al. 2009): (i) no ambiguities were removed; (ii) ambiguities were removed using a gap threshold of 0.5 and a conservation percentage of 50; and (iii) ambiguities were removed using the parameter ‘gappyout’. Phylogenetic trees for these six alignments were inferred using ModelFinder and IQ-TREE v1.6.12 (Minh et al. 2020). To quantify support for the topology, we again used 1000 ultra-fast bootstraps and 1000 SH-aLRT bootstrap replicates.

To infer the pan-SuperGroup 024 phylogeny, all amino acid sequences were aligned using both MAFFT v7.490 (Sievers et al. 2011) and MUSCLE v5.1 (Edgar 2022). Ambiguities were removed using trimAl v1.4.1 (Capella-Gutiérrez et al. 2009) and the parameter -gappyout. The phylogenetic tree was inferred using IQ-TREE v1.6.12 (Minh et al. 2020) with ModelFinder limited to LG. Support values were measured with 1000 ultra-fast bootstraps (UFboot) and 1000 sh-aLRT bootstrap replicates.

All trees were visualized with ggtree (Yu et al. 2018, Yu 2020) (implemented in R v4.4.0) and Adobe Illustrator v26.4.1.

### Motif C tally and visualization

The catalytic triad encoded by each virus in SuperGroup 024 was recorded and tabulated using R v4.4.0. The results were visualized using Prism v.10.3.0.

Sequences from individual clades were extracted from the SuperGroup 024 phylogeny by selecting individual nodes using the function ‘extract.clade()’ implemented in the R package ape. Sequences from each clade were then realigned using MAFFT and the motif C logos were generated according to the consensus sequence in Geneious Prime v2024.0.7.

## Supplementary Material

veae091_Supp

## Data Availability

All sequencing data analysed in this study are publicly available on NCBI (ormycoviruses) and an independent repository (http://47.93.21.181/, SuperGroup 024). Assembled contigs for the viruses identified in this study, the custom database used to screen libraries, alignments, and tree files are available on GitHub (https://github.com/mary-petrone/Plasmodium_ormyco).
