# Supplementary material for: A virus associated with the zoonotic pathogen Plasmodium knowlesi causing human malaria is a member of a diverse and unclassified viral taxon
Source: Virus Evol. 2024 Nov 6;10(1):veae091. doi: 10.1093/ve/veae091 (PMC11605544; doi:10.1093/ve/veae091)
Supplement: veae091_Supp [file veae091_supp.zip › suppl_data/SuppFigs.pdf]

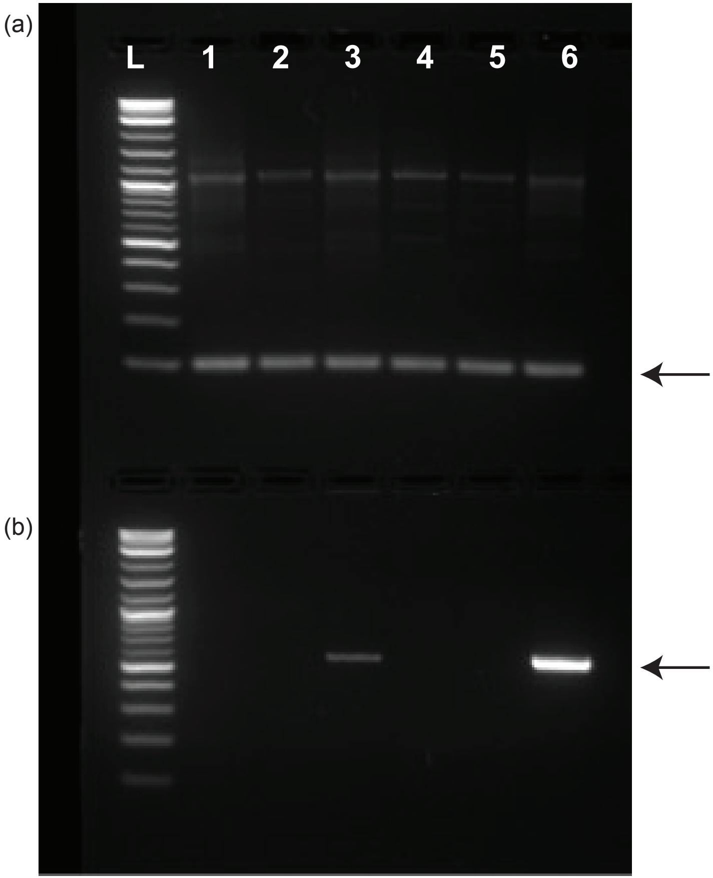

Figure S1 Detection of divergent *P. knowlesi*-associated RNA virus in two *P. knowlesi* isolates collected from human blood samples. (a) House-keeping gene *Plasmodium* LDHP (arrow). (b) Presence of the divergent RdRp segment in two *P. knowlesi* isolates (arrow).

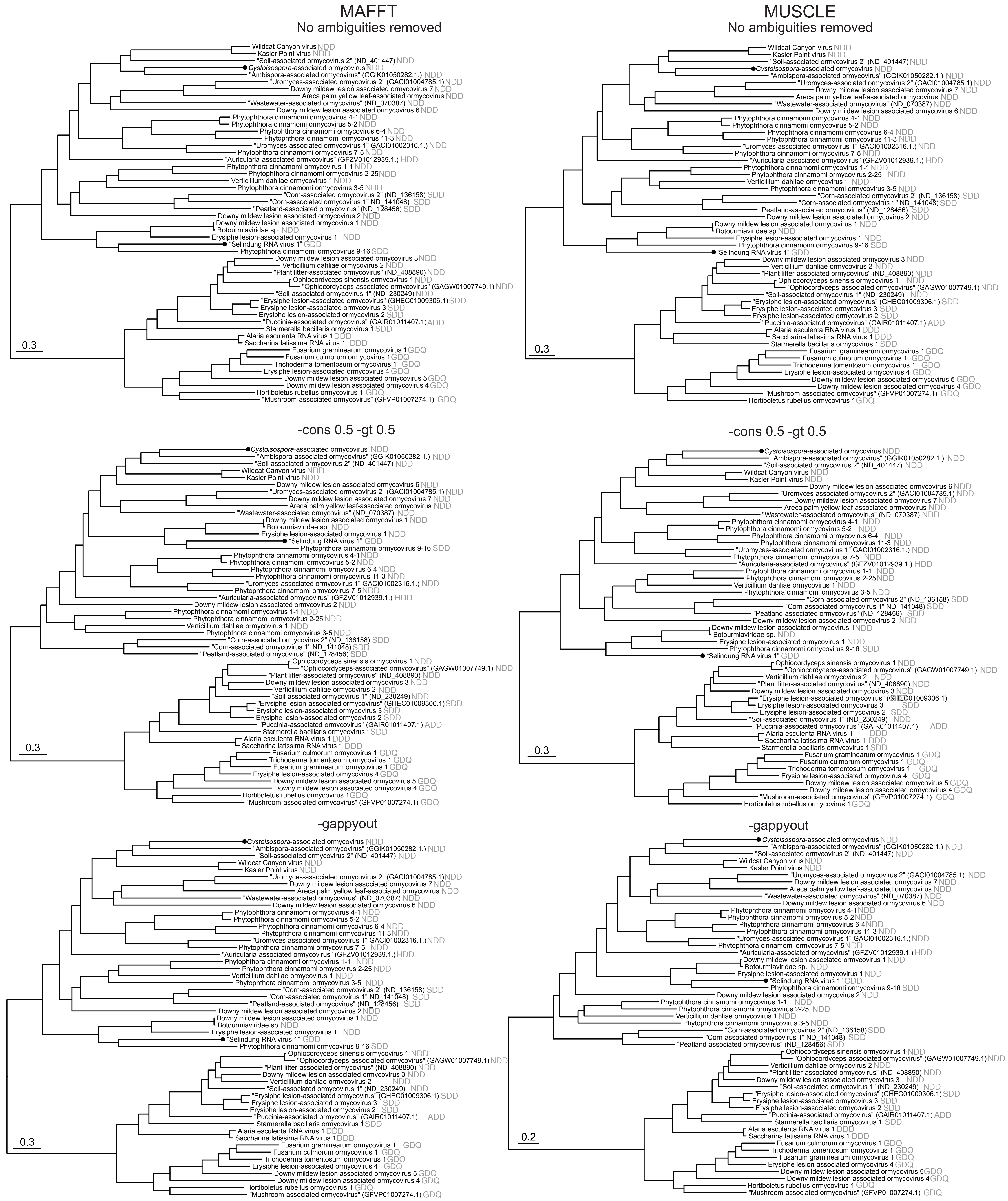

Figure S2 *Plasmodium*- and *Cystoisospera*-associated ormyco-like viruses fall within the ormycoviruses across 6 combinations of aligning and trimming methods. The Plasmodium-associated virus is denoted as "Selingdung RNA virus 1". Tree branches are scaled by number of amino acid substitutions and coloured by geographic region of sampling. Viruses identified in this study are denoted by black tips. Catalytic triads of the palm domain Motif C are shown in grey.

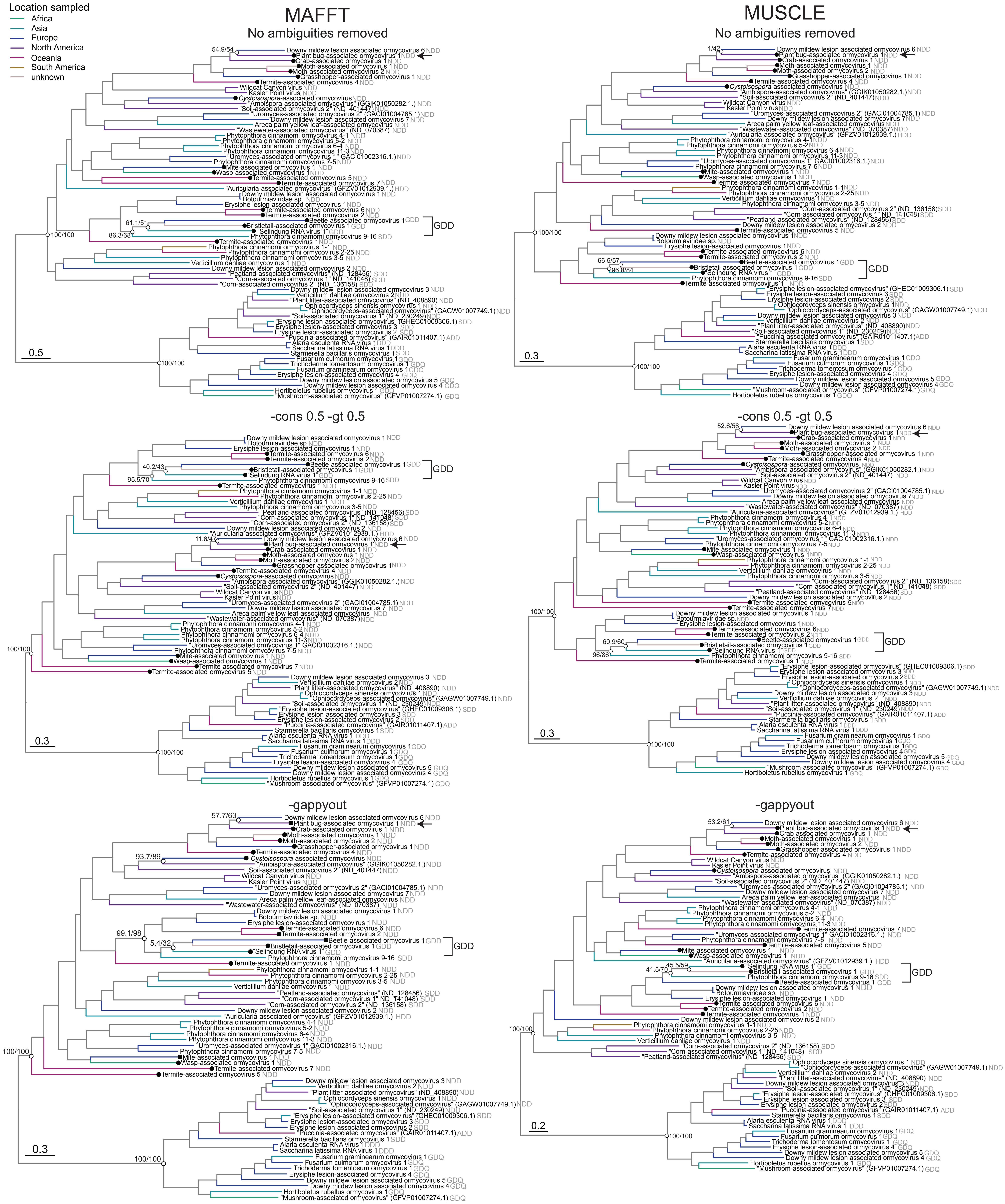

Figure S3: The topology of inferred ormycovirus phylogenetic trees is stable across 6 combinations of aligning and trimming methods. Plant bug-associated ormycovirus 1 (arrow) uses the ciliate genetic code. Support values are shown at select nodes (sh-aLRT/UFBoot). Tree branches are scaled by number of amino acid substitutions and coloured by geographic region of sampling. Viruses that encode GDD at their C motif domain are indicated with a bracket. Viruses identified in this study are denoted by black tips.

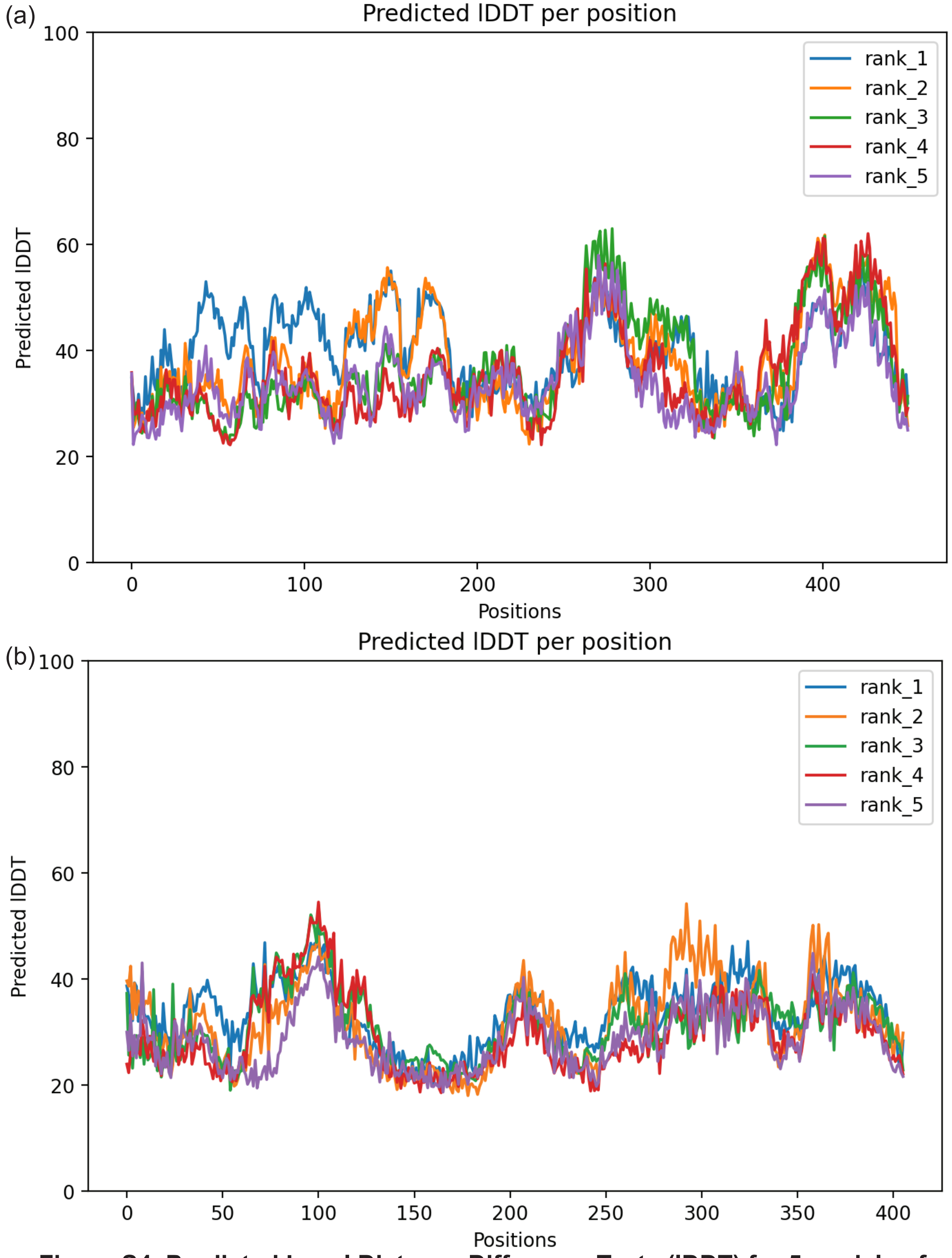

Figure S4. Predicted Local Distance Difference Tests (lDDT) for 5 models of structural predictions of the hypothetical protein of the *P. knowlesi*-associated virus (a) and Erysiphe lesion-associated ormycovirus 1 (b) generated using ColabFold.
