# Supplementary material for: A virus associated with the zoonotic pathogen Plasmodium knowlesi causing human malaria is a member of a diverse and unclassified viral taxon
Source: Virus Evol. 2024 Nov 6;10(1):veae091. doi: 10.1093/ve/veae091 (PMC11605544; doi:10.1093/ve/veae091)
Supplement: veae091_Supp [file veae091_supp.zip › suppl_data/SuppTables.pdf]

Table S1 Primers targeting the *P. knowlesi*-associated ormycovirus used for RT-PCR

Primer direction	Primer sequence
Forward	CCTGGCTTTGGGGGCAATA
Reverse	CCCATCCCTCTGGAGTCCA

Table S2 Blastx hits to P. knowelsi-associated virus

Hit name	Query cover (%)	Identity (%)	E-value
Downy mildew lesion associated ormycovirus 1 (RdRp)	49	31.78	3e-52
Botourmiaviridae sp. (RdRp) (UYL94578.1)	49	31.84	7e-52
Erysiphe lesion-associated ormycovirus 1 (RdRp)	49	32.70	4e-48
Areca palm yello leaf-associated ormycovirus (RdRp)	51	21.40	2e-04
Downy mildew lesion associated ormycovirus 2 (RdRp)	41	24.89	0.001
Wildcat Canyon virus (RdRp)	27	23.59	0.003

Table S3 Ormyco-like RdRp segments identified in 22 *Cystoisospora suis* libraries

Library	Segment Length
ERR9846864	3182
ERR9846865	3120
ERR9846866	3160
ERR9846868	3176
ERR9846869	3202
ERR9846870	3145
ERR9846872	3186
ERR9846873	3186
ERR9846874	3177
ERR9846875	3175
ERR9846876	3181
ERR9846877	3203
ERR9846878	3166
ERR9846879	3175
ERR9846880	3180
ERR9846881	3181
ERR9846882	3177
ERR9846883	3161
ERR9846884	3173
SRR4213142	3185
ERR9846867	3090
ERR9846871	3182

Table S4 Summary of ormycoviruses identified in Arthropoda TSA libraries

Contig ID	Assigned name	Sampling continent	Host taxa	RdRp length	RdRp partial/complete	Catalytic triad	Top BLASTx hit	% identity
GBHO01039923.1	Plant bug-associated ormycovirus 1	North America	Hexapoda	3277	partial	NDD	Downy mildew lesion associated ormycovirus 6	41.0
GBNA01013951.1	Wasp-associated ormycovirus 1	Africa	Hexapoda	3056	complete	NDD	Phytophthora cinnamomi ormycovirus 6-4	30.7
GBTA01002218.1	Moth-associated ormycovirus 2	Oceania	Hexapoda	801	partial	NDD	Downy mildew lesion associated ormycovirus 6	38.8
GBTA01054149.1	Moth-associated ormycovirus 1	Oceania	Hexapoda	3801	partial	NDD	Downy mildew lesion associated ormycovirus 6	34.5
GDXN01051862.1	Grasshopper-associated ormycovirus 1	Europe	Hexapoda	1475	partial	NDD	Downy mildew lesion associated ormycovirus 6	35.1
GEYJ01092710.1	Mite-associated ormycovirus 1	Europe	Chelicerata	2589	partial	NDD	Phytophthora cinnamomi ormycovirus 6-4	32.3
GFJG01059483.1	Crab-associated ormycovirus 1	North America	Crustacea	3396	partial	NDD	Downy mildew lesion associated ormycovirus 6	40.0
GHZM01086934.1	Termite-associated ormycovirus 6	Oceania	Hexapoda	1223	partial	NDD	Erysiphe lesion-associated ormycovirus 1	35.5
GHZM01115244.1	Termite-associated ormycovirus 7	Oceania	Hexapoda	2814	partial	NDD	Phytophthora cinnamomi ormycovirus 11-3	29.0
GHZM01131186.1	Termite-associated ormycovirus 5	Oceania	Hexapoda	2977	partial	NDD	Phytophthora cinnamomi ormycovirus 7-5	27.4
GHZM01206470.1	Termite-associated ormycovirus 4	Oceania	Hexapoda	2785	partial	NDD	Downy mildew lesion associated ormycovirus 6	34.4
GHZM01445935.1	Termite-associated ormycovirus 2	Oceania	Hexapoda	2756	partial	NDD	Botourmiaviridae sp. (UYL94578)	32.0
GIAM01545236.1	Termite-associated ormycovirus 1	Oceania	Hexapoda	2903	partial	NDD	Erysiphe lesion-associated ormycovirus 1	27.1
GKFO01000268.1	Beetle-associated ormycovirus 1	Europe	Hexapoda	1082	partial	GDD	Downy mildew lesion associated ormycovirus 1	28.2
HBDP01107766.1	Bristletail-associated ormycovirus 1	unknown	Hexapoda	3120	complete	GDD	Erysiphe lesion-associated ormycovirus 1	29.0
